# Supplementary material for: Joint modeling of an outcome variable and integrated omics datasets using GLM-PO2PLS
Source: J Appl Stat. 2024 Feb 21;51(13):2627–51. doi: 10.1080/02664763.2024.2313458 (PMC11404385; doi:10.1080/02664763.2024.2313458)
Supplement: Supplemental Material [file CJAS_A_2313458_SM2803.pdf]

Supplementary Materials for Joint Modeling of An Outcome Variable and Integrated Omics Datasets Using GLM-PO2PLS

Zhujie Gu[1,2], Hae-Won Uh[1], Jeanine Houwing-Duistermaat[3,1,4], and Said el Bouhaddani[1]

[1]Dept. of Data Science and Biostatistics, Julius Centre, UMC Utrecht, The Netherlands
[2]Medical Research Council Biostatistics Unit, University of Cambridge, UK
[3]Dept. of Statistics, University of Leeds, UK
[4]Dept. of Mathematics, Radboud University, Nijmegen, The Netherlands

This document is supplement to the main article, "Joint Modeling of An Outcome Variable and Integrated Omics Datasets Using GLM-PO2PLS". It is structured into two parts: Methods, and Simulation results. Each part contains additional materials for the respective section in the main article.

In section S 1, we first give mathematical details for the EM algorithms for both GLM-PO2PLS continuous and binary models. We then prove the asymptotic normality of the estimator, and give equations for the observed Fisher information matrix needed in calculating the test statistics. In section S 2, we show simulation results omitted in the main article and the additional simulations.

S 1 Methods

S 1.1 An EM algorithm for GLM-PO2PLS with a normally distributed outcome

Let X, Y and Z be data matrices consisting of N observations of (x, y, z). For empirical identifiability of the components, we assume $\max(r + K_x, r + K_y) < N$.

In the E step, the expectation of the complete data log likelihood for one subject can be decomposed to factors that depend on distinct sets of parameters as follows,

$$\begin{aligned} Q(\theta|\theta') =& \mathbb{E}[\log f(x, y, z, t, u, t_\perp, u_\perp)] = \mathbb{E}[\log f(x, y, z|t, u, t_\perp, u_\perp)] + \mathbb{E}[\log f(t, u, t_\perp, u_\perp)] \\ =& \underbrace{\mathbb{E}[\log f(z|t, u)]}_{Q_{\{a,b,\sigma_g^2\}}} + \underbrace{\mathbb{E}[\log f(x|t, t_\perp)]}_{Q_{\{W,W_\perp,\sigma_e^2\}}} + \underbrace{\mathbb{E}[\log f(y|u, u_\perp)]}_{Q_{\{C,C_\perp,\sigma_f^2\}}} \\ &+ \underbrace{\mathbb{E}[\log f(u|t)]}_{Q_{\{B,\Sigma_h\}}} + \underbrace{\mathbb{E}[\log f(t)]}_{Q_{\Sigma_t}} + \underbrace{\mathbb{E}[\log f(t_\perp)]}_{Q_{\Sigma_{t_\perp}}} + \underbrace{\mathbb{E}[\log f(u_\perp)]}_{Q_{\Sigma_{u_\perp}}}. \end{aligned} \quad (1)$$

In this equation, the conditioning on x, y, z and θ' is dropped, to simplify notation. Given the observed data for N subjects, the factorized conditional expectations in (1) are calculated as

follows. Let $(T, U, T_\perp, U_\perp)$ be the collection of row vectors $(t, u, t_\perp, u_\perp)$ for N subjects.

$$
\begin{aligned}
Q_{\{a,b,\sigma_g^2\}} &= -\frac{1}{2}\left\{ N\log(2\pi\sigma_g^2) + \frac{1}{\sigma_g^2}\operatorname{tr}\mathbb{E}\left[(Z - Ta^\top - (U-TB)b^\top)^\top (Z - Ta^\top - (U-TB)b^\top)\right] \right\},\\
Q_{\{W,W_\perp,\sigma_e^2\}} &= -\frac{1}{2}\left\{ Np\log(2\pi\sigma_e^2) + \frac{1}{\sigma_e^2}\operatorname{tr}\mathbb{E}\left[(X - TW^\top - T_\perp W_\perp^\top)^\top (X - TW^\top - T_\perp W_\perp^\top)\right] \right\},\\
Q_{\{C,C_\perp,\sigma_f^2\}} &= -\frac{1}{2}\left\{ Nq\log(2\pi\sigma_f^2) + \frac{1}{\sigma_f^2}\operatorname{tr}\mathbb{E}\left[(Y - UC^\top - U_\perp C_\perp^\top)^\top (Y - UC^\top - U_\perp C_\perp^\top)\right] \right\},\\
Q_{\{B,\Sigma_h\}} &= -\frac{1}{2}\left\{ NK\log(2\pi) + N\log|\Sigma_h| + \operatorname{tr}\mathbb{E}\left[(U-TB)^\top (U-TB)\Sigma_h^{-1}\right] \right\},\\
Q_{\Sigma_t} &= -\frac{1}{2}\left\{ NK\log(2\pi) + N\log|\Sigma_t| + \operatorname{tr}\mathbb{E}\left[T^\top T\Sigma_t^{-1}\right] \right\},\\
Q_{\Sigma_{t_\perp}} &= -\frac{1}{2}\left\{ NK_x\log(2\pi) + N\log|\Sigma_{t_\perp}| + \operatorname{tr}\mathbb{E}\left[T_\perp^\top T_\perp\Sigma_{t_\perp}^{-1}\right] \right\},\\
Q_{\Sigma_{u_\perp}} &= -\frac{1}{2}\left\{ NK_y\log(2\pi) + N\log|\Sigma_{u_\perp}| + \operatorname{tr}\mathbb{E}\left[U_\perp^\top U_\perp\Sigma_{u_\perp}^{-1}\right] \right\}.
\end{aligned}
\tag{2}
$$

Here, the conditional expectations involve the first and second conditional moments of the vector $(t, u, t_\perp, u_\perp)$ given x, y, z and θ' for each subject. Since the complete data vector for one subject $(x, y, z, t, u, t_\perp, u_\perp)$ follows a multivariate normal distribution with zero mean and known covariance matrix, the conditional distribution $(t, u, t_\perp, u_\perp | x, y, z)$ for each subject can be calculated explicitly following lemma 3 in [3] as follows:

$$(t, u, t_\perp, u_\perp | x, y, z) \sim \mathcal{N}\left((x, y, z)\Sigma_\epsilon \Gamma \tilde{\Sigma}_m, \tilde{\Sigma}_m\right),$$

where

$$
\begin{aligned}
\tilde{\Sigma}_m &= \{\Sigma_m^{-1} + \Gamma^\top \Sigma_\epsilon^{-1}\Gamma\}^{-1},\\
\Sigma_\epsilon &= \begin{bmatrix} I_p\sigma_e^2 & 0 & 0\\ 0 & I_q\sigma_f^2 & 0\\ 0 & 0 & \sigma_g^2 \end{bmatrix},\\
\Gamma &= \begin{bmatrix} W & 0 & W_\perp & 0\\ 0 & C & 0 & C_\perp\\ a - bB & b & 0 & 0 \end{bmatrix},\\
\Sigma_m &= \begin{bmatrix} \Sigma_t & \Sigma_t B & 0 & 0\\ \Sigma_t B & \Sigma_u & 0 & 0\\ 0 & 0 & \Sigma_{t_\perp} & 0\\ 0 & 0 & 0 & \Sigma_{u_\perp} \end{bmatrix}.
\end{aligned}
$$

In the M step, we set the partial derivatives of the conditional expectations in (2) to zero and get an update of each parameter.

Regarding the first term $Q_{\{a,b,\sigma_g^2\}}$, taking partial derivatives with respect to $\alpha = (a, b)$ yields

$$\hat{\alpha} = Z^\top \mathbb{E}[(T, H)]\, \mathbb{E}[(T, H)^\top (T, H)]^{-1}.$$

This resembles the usual maximum likelihood estimator for the regression coefficient in a linear regression model where Z is regressed on $\mathbb{E}[(T, H)]$. Taking partial derivatives with respect to σ_g^2 yields the well-known maximum likelihood estimator for the residual variance

$$\hat{\sigma_g^2} = \frac{1}{N}\operatorname{tr}\mathbb{E}[(Z - (T, H)(a, b)^\top)^\top (Z - (T, H)(a, b)^\top)] = \frac{1}{N}\operatorname{tr}\mathbb{E}[G^\top G].$$

Regarding $Q_{\{W,W_\perp,\sigma_e^2\}}$ which involves optimization over semi-orthogonal loading matrices W and $W_\perp$, Lagrange multipliers Λ_W and $\Lambda_{W_\perp}$ are introduced. The objective function to minimize is then

$$\mathrm{tr}\,\mathbb{E}\left[(X - TW^\top - T_\perp W_\perp^\top)^\top (X - TW^\top - T_\perp W_\perp^\top)\right] + \Lambda_W(W^\top W - I_K) + \Lambda_{W_\perp}(W_\perp^\top W_\perp - I_{K_x}). \tag{3}$$

Note that the objective function involves both W and $W_\perp$ and cannot be decoupled. We adopt here the same strategy used in [3] that performs sequential optimization [7]. First, (3) is minimized over W, keeping $W_\perp$ constant,

$$\hat{W} = (X^\top \mathbb{E}[T] - W_\perp \mathbb{E}[T_\perp^\top T])(E[T^\top T] + \Lambda_W)^{-1} = \mathrm{orth}(X^\top \mathbb{E}[T] - W_\perp \mathbb{E}[T_\perp^\top T]), \tag{4}$$

where $\mathrm{orth}(A) = JV^\top$ with J and V the singular vectors of A. The last step is proven in [2]. Next, (3) is optimized over $W_\perp$, keeping W equal to its minimizer,

$$\hat{W_\perp} = (X^\top \mathbb{E}[T_\perp] - \hat{W}\,\mathbb{E}[T^\top T_\perp])(E[T_\perp^\top T_\perp] + \Lambda_{W_\perp})^{-1} = \mathrm{orth}(X^\top \mathbb{E}[T_\perp] - \hat{W}\,\mathbb{E}[T^\top T_\perp]).$$

In the same way, estimates for semi-orthogonal loading matrices C and $C_\perp$ can be obtained.

For the matrices that are restricted to be diagonal, for example, the inner regression matrix B, we set the off-diagonals to zero using its Hadamard product with an identity matrix as follows,

$$\hat{B} = \mathbb{E}[U^\top T](\mathbb{E}[T^\top T])^{-1} \circ I_K.$$

Now the EM updates at step k can be written as follows, starting with an initial guess for $k = 0$. Denote $\mathbb{E}_k[\cdot] = \mathbb{E}[\cdot|X, Y, Z, \theta^k]$.

$$\begin{aligned}
(a,b)^{k+1} &= Z^\top \mathbb{E}_k[(T,H)]\mathbb{E}_k[(T,H)^\top (T,H)]^{-1} \\
W^{k+1} &= \mathrm{orth}(X^\top \mathbb{E}_k[T] - W_\perp^k \mathbb{E}_k[T_\perp^\top T]) \\
W_\perp^{k+1} &= \mathrm{orth}(X^\top \mathbb{E}_k[T_\perp] - W^{k+1}\mathbb{E}_k[T^\top T_\perp]) \\
C^{k+1} &= \mathrm{orth}(Y^\top \mathbb{E}_k[U] - C_\perp^k \mathbb{E}_k[U_\perp^\top U]) \\
C_\perp^{k+1} &= \mathrm{orth}(Y^\top \mathbb{E}_k[U_\perp] - C^{k+1}\mathbb{E}_k[U^\top U_\perp]) \\
B^{k+1} &= \mathbb{E}_k[U^\top T](\mathbb{E}_k[T^\top T])^{-1} \circ I_K \\
\Sigma_t^{k+1} &= \frac{1}{N}\mathbb{E}_k[T^\top T] \circ I_K \\
\Sigma_{t_\perp}^{k+1} &= \frac{1}{N}\mathbb{E}_k[T_\perp^\top T_\perp] \circ I_{K_x} \\
\Sigma_{u_\perp}^{k+1} &= \frac{1}{N}\mathbb{E}_k[U_\perp^\top U_\perp] \circ I_{K_y} \\
\Sigma_h^{k+1} &= \frac{1}{N}\mathbb{E}_k[H^\top H] \circ I_K \\
(\sigma_e^2)^{k+1} &= \frac{1}{Np}\,\mathrm{tr}(\mathbb{E}_k[E^\top E]) \\
(\sigma_f^2)^{k+1} &= \frac{1}{Nq}\,\mathrm{tr}(\mathbb{E}_k[F^\top F]) \\
(\sigma_g^2)^{k+1} &= \frac{1}{N}\,\mathrm{tr}(\mathbb{E}_k[G^\top G])
\end{aligned} \tag{5}$$

S 1.2 An EM algorithm for GLM-PO2PLS model with a Bernoulli distributed outcome

The associated log-likelihood for the GLM-PO2PLS model with a Bernoulli distributed outcome involves an integral with respect to $(\nu, \xi) = ((t,u),(t_\perp,u_\perp))$ of dimension $(2K + K_x + K_y)$. By

integrating out ξ, the dimension of integral can be reduced to $2K$ as follows

$$\ell(\theta; x, y, z) = \log \int_{(\nu,\xi)} f(x, y, z|\nu, \xi, \theta) f(\nu, \xi|\theta) d(\nu, \xi) = \log \int_{\nu} p(z|\nu) f(x|\nu) f(y|\nu) f(\nu) d\nu.$$

The conditional probability mass/density functions involved are given by

$$\begin{aligned}
p(z|\nu) &= \begin{cases} \Big(1 + \exp\{-(a_0 + ta^\top + (u - tB)b^\top)\}\Big)^{-1}, & z = 1 \\ \Big(1 + \exp\{a_0 + ta^\top + (u - tB)b^\top\}\Big)^{-1}, & z = 0, \end{cases} \\
f(x|\nu) &= (2\pi)^{-\frac{p}{2}} |\Sigma_{x|t}|^{-\frac{1}{2}} exp\Big(- \frac{1}{2}(x - tW^\top)\Sigma_{x|t}^{-1}(x - tW^\top)^\top \Big), \\
f(y|\nu) &= (2\pi)^{-\frac{q}{2}} |\Sigma_{y|u}|^{-\frac{1}{2}} exp\Big(- \frac{1}{2}(y - uC^\top)\Sigma_{y|u}^{-1}(y - uC^\top)^\top \Big), \\
f(\nu) &= (2\pi)^{-K} |\Sigma_\nu|^{-\frac{1}{2}} exp\Big(- \frac{1}{2}\nu\Sigma_\nu^{-1}\nu^\top \Big),
\end{aligned} \tag{6}$$

where

$$\Sigma_{x|t} = W_\perp \Sigma_{t_\perp} W_\perp^\top + \sigma_e^2 I_p, \qquad \Sigma_{y|u} = C_\perp \Sigma_{u_\perp} C_\perp^\top + \sigma_f^2 I_q, \qquad \Sigma_\nu = \begin{bmatrix} \Sigma_t & \Sigma_t B \\ B\Sigma_t & \Sigma_u \end{bmatrix}.$$

Note here that the determinant and the inverse of a $p \times p$ matrix $\Sigma_{x|t}$ (and a $q \times q$ matrix $\Sigma_{y|u}$) are required in (6). Calculating them directly is not feasible, even for a moderate p (or q). Following matrix determinant lemma [4] and Woodbury matrix identity [6], we perform the following transformation, such that only calculation of the determinant and inverse of a $K_x \times K_x$ (or $K_y \times K_y$) matrix is required. Here, the transformation utilizes the semi-orthogonality constraint of $W_\perp^\top W_\perp = I_{K_x}$.

$$\begin{aligned}
\log |\Sigma_{x|t}| &= \log \Big| I_{K_x} + \frac{1}{\sigma_e^2}\Sigma_{t_\perp} \Big| + p \log \sigma_e^2, \\
\Sigma_{x|t}^{-1} &= \frac{1}{\sigma_e^2}\Big(I_p - \frac{1}{\sigma_e^2} W_\perp (\Sigma_{t_\perp}^{-1} + \frac{1}{\sigma_e^2} I_{K_x})^{-1} W_\perp^\top \Big),
\end{aligned}$$

The determinant and inverse of $\Sigma_{y|u}$ are calculated analogously.

In the EM algorithm, we consider the partial complete data vector (x, y, z, ν). For each current estimate θ', the algorithm optimizes for each subject the objective function

$$Q(\theta|\theta') = \mathbb{E}[\log f(x, y, z, \nu|\theta)|x, y, z, \theta']. \tag{7}$$

Similar to (1), the conditional expectation in (7) can be decomposed to factors that depend on distinct sets of parameters,

$$\begin{aligned}
Q(\theta|\theta') &= \mathbb{E}[\log f(x, y, z, \nu)] = \mathbb{E}[\log f(x, y, z|\nu)] + \mathbb{E}[\log f(\nu)] \\
&= \underbrace{\mathbb{E}[\log p(z|\nu)]}_{Q_{\{a_0,a,b\}}} + \underbrace{\mathbb{E}[\log f(x|t)]}_{Q_{\{W,W_\perp,\sigma_e^2,\Sigma_{t_\perp}\}}} + \underbrace{\mathbb{E}[\log f(y|u)]}_{Q_{\{C,C_\perp,\sigma_f^2,\Sigma_{u_\perp}\}}} + \underbrace{\mathbb{E}[\log f(u|t)]}_{Q_{\{B,\Sigma_h\}}} + \underbrace{\mathbb{E}[\log f(t)]}_{Q_{\Sigma_t}}.
\end{aligned} \tag{8}$$

The first term $Q_{\{a_0,a,b\}}$ needs to be estimated with numerical integration for each observed instance (X_i, Y_i, Z_i) with respect to $\nu_i = (T_i, U_i)$ as follows,

$$Q_{\{a_0,a,b\}} = \sum_{i=1}^{N} \int [\log p(Z_i|\nu_i)] f(\nu_i|X_i, Y_i, Z_i, \theta') d\nu_i = \sum_{i=1}^{N} \frac{\int [\log p(Z_i|\nu_i)] p(Z_i|\nu) f(X_i|\nu_i) f(Y_i|\nu_i) f(\nu_i) d\nu_i}{\int p(Z_i|\nu_i) f(X_i|\nu_i) f(Y_i|\nu_i) f(\nu_i) d\nu_i}. \tag{9}$$

The numerator and denominator for each subject in (9) can be approximated separately by

$$\int \varphi(\nu)p(z|\nu)f(x,y|\nu)f(\nu)d\nu \approx \sum_{m_1=1}^{M} \cdots \sum_{m_{2r}=1}^{M} \varphi(\nu=\nu_m)p(z|\nu=\nu_m)f(x,y|\nu=\nu_m)w_{m_1}\dots w_{m_{2r}}, \tag{10}$$

with the function φ being $\log p(z|\nu)$ and 1, respectively.

Given the observed data for N subjects, the other terms in (8) are given by

$$\begin{aligned}
Q_{\{W,W_\perp,\sigma_e^2,\Sigma_{t_\perp}\}} &= -\frac{1}{2}\left(Np\log(2\pi) + N\log|\Sigma_{x|t}| + \operatorname{tr}\mathbb{E}\left[(X-TW^\top)^\top \Sigma_{x|t}^{-1}(X-TW^\top)\right]\right),\\
Q_{\{C,C_\perp,\sigma_f^2,\Sigma_{u_\perp}\}} &= -\frac{1}{2}\left(Nq\log(2\pi) + N\log|\Sigma_{y|u}| + \operatorname{tr}\mathbb{E}\left[(Y-UC^\top)^\top \Sigma_{y|u}^{-1}(Y-UC^\top)\right]\right),\\
Q_{\{B,\Sigma_h\}} &= -\frac{1}{2}\left\{NK\log(2\pi) + N\log|\Sigma_h| + \operatorname{tr}\mathbb{E}\left[(U-TB)^\top(U-TB)\Sigma_h^{-1}\right]\right\},\\
Q_{\Sigma_t} &= -\frac{1}{2}\left\{NK\log(2\pi) + N\log|\Sigma_t| + \operatorname{tr}\mathbb{E}\left[T^\top T\Sigma_t^{-1}\right]\right\}.
\end{aligned} \tag{11}$$

The conditional expectations in (11) involve calculation of the first and second conditional moments of $\nu_i = (T_i, U_i)$ for each subject i. The conditional moments are given by

$$\begin{aligned}
\mathbb{E}[\nu_i|X_i,Y_i,Z_i,\theta'] &= \frac{\int \nu_i p(Z_i|\nu_i)f(X_i|\nu_i)f(Y_i|\nu_i)f(\nu_i)d\nu_i}{\int p(Z_i|\nu_i)f(X_i|\nu_i)f(Y_i|\nu_i)f(\nu_i)d\nu_i}\\
\mathbb{E}[\nu_i^\top\nu_i|X_i,Y_i,Z_i,\theta'] &= \frac{\int \nu_i^\top\nu_i p(Z_i|\nu_i)f(X_i|\nu_i)f(Y_i|\nu_i)f(\nu_i)d\nu_i}{\int p(Z_i|\nu_i)f(X_i|\nu_i)f(Y_i|\nu_i)f(\nu_i)d\nu_i}
\end{aligned}$$

Here, the integrals are numerically calculated with (10).

In the M step, maximizing $Q_{\{a_0,a,b\}}$ requires iteration. We propose a one-step gradient descent strategy to find an update of $\beta = (a_0, a, b)$ along the direction of the gradient given by

$$\nabla Q_\beta = \sum_{i=1}^{N}\left\{\frac{\int [\frac{\partial}{\partial\beta}\log p(Z_i|\nu_i)]p(Z_i|\nu)f(X_i|\nu_i)f(Y_i|\nu_i)f(\nu_i)d\nu_i}{\int p(Z_i|\nu_i)f(X_i|\nu_i)f(Y_i|\nu_i)f(\nu_i)d\nu_i}\right\}^\top.$$

A step size that guarantees the increase of Q_β is searched using the backtracking rule [1].

To estimate the semi-orthogonal joint loading matrix W, we relax the orthogonality constraint temporarily, and obtain an intermediate estimator $\hat{W}_*$ by setting the partial derivative of $Q_{\{W,W_\perp,\sigma_e^2,\Sigma_{t_\perp}\}}$ with respect to W to zero,

$$\hat{W}_* = X^\top\,\mathbb{E}[T]\,\mathbb{E}[T^\top T]^{-1}.$$

To impose the orthogonality constraint, the "orth" operator in (4) is used,

$$\hat{W} = \operatorname{orth}(\hat{W}_*).$$

This strategy was also used in [5] for estimation of orthogonal loading matrices.

The parameters $W_\perp, \sigma_e^2, \Sigma_{t_\perp}$ are contained in $\Sigma_{x|t}$. Therefore, we take derivative of $Q_{\{W,W_\perp,\sigma_e^2,\Sigma_{t_\perp}\}}$ with respect to $\Sigma_{x|t}$ as follows,

$$\frac{\partial Q_{\{W,W_\perp,\sigma_e^2,\Sigma_{t_\perp}\}}}{\partial\Sigma_{x|t}} = \Sigma_{x|t}^{-1} - \frac{1}{N}\Sigma_{x|t}^{-1}\,\mathbb{E}[(X-TW^\top)^\top(X-TW^\top)]\Sigma_{x|t}^{-1}. \tag{12}$$

Since $\Sigma_{x|t} = W_\perp \Sigma_{t_\perp} W_\perp^\top + \sigma_e^2 I_p$ is a full-rank matrix, by setting (12) to zero, we get the following relationship,

$$\frac{1}{N}\mathbb{E}[(X - TW^\top)^\top (X - TW^\top)] = \Sigma_{x|t} = W_\perp \Sigma_{t_\perp} W_\perp^\top + \sigma_e^2 I_p.$$

Taking trace of both sides, σ_e^2 can be estimated as

$$\hat{\sigma_e^2} = \frac{1}{p}(\frac{1}{N}\operatorname{tr}\mathbb{E}[(X - TW^\top)^\top (X - TW^\top)] - \operatorname{tr}[\Sigma_{t_\perp}]).$$

Note that $W_\perp$ and $\Sigma_{t_\perp}$ can be obtained by eigendecomposition of the real symmetric matrices $(\frac{1}{N}\mathbb{E}[(X - TW^\top)^\top (X - TW^\top)] - \hat{\sigma_e^2} I_p)$. Here, power iteration is used to avoid processing a $p \times p$ matrix. Parameters in $Q_{\{C, C_\perp, \sigma_f^2, \Sigma_{u_\perp}\}}$ are estimated analogously.

Using the same notation as in (5), the EM algorithm updates parameters in step k as follows:

$$\begin{aligned}
(a0, a, b)^{k+1} &= (a0, a, b)^k + s^{k+1} \nabla Q_\beta^{k+1} \\
W^{k+1} &= \operatorname{orth}(X^\top \mathbb{E}_k[T] \mathbb{E}_k[T^\top T]^{-1}) \\
C^{k+1} &= \operatorname{orth}(Y^\top \mathbb{E}_k[U] \mathbb{E}_k[U^\top U]^{-1}) \\
B^{k+1} &= \mathbb{E}_k[U^\top T](\mathbb{E}_k[T^\top T])^{-1} \circ I_K \\
\Sigma_t^{k+1} &= \frac{1}{N}\mathbb{E}_k[T^\top T] \circ I_K \\
\Sigma_h^{k+1} &= \frac{1}{N}\mathbb{E}_k[H^\top H] \circ I_K \\
(\sigma_e^2)^{k+1} &= \frac{1}{p}(\frac{1}{N}\operatorname{tr}\mathbb{E}_k[(X - TW^\top)^\top (X - TW^\top)] - \operatorname{tr}[\Sigma_{t_\perp}^k]) \\
(\sigma_f^2)^{k+1} &= \frac{1}{q}(\frac{1}{N}\operatorname{tr}\mathbb{E}_k[(Y - UC^\top)^\top (Y - UC^\top)] - \operatorname{tr}[\Sigma_{u_\perp}^k]) \\
W_\perp^{k+1} &= \text{eigen vectors of } (\frac{1}{N}\mathbb{E}_k[(X - TW^{k+1^\top})^\top (X - TW^{k+1^\top})] - (\sigma_e^2)^{k+1} I_p) \\
C_\perp^{k+1} &= \text{eigen vectors of } (\frac{1}{N}\mathbb{E}_k[(Y - UC^{k+1^\top})^\top (Y - UC^{k+1^\top})] - (\sigma_f^2)^{k+1} I_q) \\
\Sigma_{t_\perp}^{k+1} &= \operatorname{diag}[\text{eigen values of } (\frac{1}{N}\mathbb{E}_k[(X - TW^{k+1^\top})^\top (X - TW^{k+1^\top})] - (\sigma_e^2)^{k+1} I_p)] \\
\Sigma_{u_\perp}^{k+1} &= \operatorname{diag}[\text{eigen values of } (\frac{1}{N}\mathbb{E}_k[(Y - UC^{k+1^\top})^\top (Y - UC^{k+1^\top})] - (\sigma_f^2)^{k+1} I_q)]
\end{aligned}$$

S 1.3 The asymptotic distribution

Recall the GLM-PO2PLS model with a normally distributed outcome,

$$x = tW^\top + t_\perp W_\perp^\top + e, \quad y = uC^\top + u_\perp C_\perp^\top + f, \quad u = tB + h,$$
$$z = ta^\top + ub^\top + g.$$

The parameters are collected in $\theta = \{W, C, W_\perp, C_\perp, a, b, B, \Sigma_t, \Sigma_{t_\perp}, \Sigma_{u_\perp}, \sigma_e^2, \sigma_f^2, \Sigma_h, \sigma_g^2\}$, and the associated log-likelihood is given by

$$\ell(\theta; x, y, z) = -\frac{1}{2}\{(p + q + 1)\log(2\pi) + \log|\Sigma_\theta| + (x, y, z)\Sigma_\theta^{-1}(x, y, z)^\top\}.$$

Here, the covariance matrix Σ_θ is

$$\Sigma_\theta = \begin{bmatrix} W\Sigma_t W^\top + W_\perp \Sigma_{t_\perp} W_\perp^\top + \sigma_e^2 I_p & W\Sigma_t B C^\top & W\Sigma_t a^\top \\ CB\Sigma_t W^\top & C\Sigma_u C^\top + C_\perp \Sigma_{u_\perp} C_\perp^\top + \sigma_f^2 I_q & C(\Sigma_h b^\top + B\Sigma_t a^\top) \\ a\Sigma_t W^\top & (a\Sigma_t B + b\Sigma_h)C^\top & a\Sigma_t a^\top + b\Sigma_h b^\top + \sigma_g^2 \end{bmatrix}. \tag{13}$$

We show here that under certain regularity conditions, consistency of the estimator θ and its asymptotic distribution $\mathcal{N}(\theta, \Pi_\theta)$ follows from Shapiro's Proposition 4.2 applies to the GLM-PO2PLS-Normal model. It has been shown in [3] that the proposition applies to the PO2PLS model. Similarly, we define τ as the mapping from a θ' to $\Sigma_{\theta'}$, given in (13), and the discrepancy function F as $F(S;\Sigma_{\theta'}) = N\log|\Sigma_{\theta'}| + \operatorname{tr} S\Sigma_{\theta'}^{-1} - N\log|S| + \operatorname{tr} SS^{-1}$, where S is the maximum likelihood estimator of the covariance matrix of (x, y, z). The function F can be recognized as the discrepancy of two log-likelihoods evaluated at S and $\Sigma_{\theta'}$, respectively, with a minimizer $\Sigma_{\hat{\theta}}$. The mapping function τ is analytic and quadratic in θ'. It follows from the definition of F and the regularity of the normal log-likelihood ℓ that F is non-negative, zero only if $S = \Sigma_{\theta'}$, and positive everywhere else. Also, ℓ, thus F, is twice continuously differentiable and since GLM-PO2PLS is identifable, F has a positive definite Hessian at θ'. Then, Proposition 4.2 in Shapiro (1986) states that the elements of $\Sigma_{\hat{\theta}}$ are asymptotically normally distributed. Theorem 2 in the main article follows,

$$N^{1/2}(\hat{\theta} - \theta) \longrightarrow \mathcal{N}(0, \Pi_\theta).$$

Covariance matrix of the coefficients Let $\alpha = (a, b)$ and $\alpha_k = (a_k, b_k)$. The two test statistics $T_{full} = \hat{\alpha}\Pi_{\hat{\alpha}}^{-1}\hat{\alpha}^\top$, and $T_{comp.wise} = \hat{\alpha}_k\Pi_{\hat{\alpha}_k}^{-1}\hat{\alpha}_k{}^\top$ involve calculation of the covariance matrix of the coefficients $\Pi_{\hat{\alpha}}$.

Let $\mathcal{I}(\hat{\theta})$ be the observed Fisher information matrix. To obtain $\Pi_{\hat{\alpha}}$, the submatrix of $\mathcal{I}^{-1}(\hat{\theta})$ corresponding to $\hat{\alpha}$ (denote $\mathcal{I}^{-1}(\hat{\alpha})$) has to be calculated. However, inverting $\mathcal{I}(\hat{\theta})$ is computationally infeasible, even for moderate dimensions. Under additional assumptions that $\hat{\alpha}$ and $\hat{\theta}/\hat{\alpha}$ are asymptotically independent and $\hat{\sigma}_g^2$ is non-random, $\mathcal{I}^{-1}(\hat{\alpha})$ can be calculated, and be used to approximate $\Pi_{\hat{\alpha}}$. The $2K \times 2K$ observed Fisher information matrix $\mathcal{I}(\hat{\alpha})$ is given by

$$\begin{aligned}\mathcal{I}(\hat{\alpha}) &= \sum_{i=1}^{N} \mathbb{E}[B_i(\hat{\alpha})|\psi_i] - \sum_{i=1}^{N}\sum_{j=1}^{N} \mathbb{E}[S_i(\hat{\alpha})S_j(\hat{\alpha})^\top|\psi_i;\psi_j] \\ &= \sum_{i=1}^{N} \mathbb{E}[B_i(\hat{\alpha})|\psi_i] - \sum_{i=1}^{N} \mathbb{E}[S_i(\hat{\alpha})S_i(\hat{\alpha})^\top|\psi_i] - \sum_{i=1}^{N}\sum_{j=1, j\neq i}^{N} \mathbb{E}[S_i(\hat{\alpha})|\psi_i]\,\mathbb{E}[S_j(\hat{\alpha})|\psi_j]^\top\end{aligned} \tag{14}$$

Here, $S_i(\hat{\alpha}) = \frac{1}{\hat{\sigma_g^2}}\left((T_i, H_i)^\top Z_i - (T_i, H_i)^\top (T_i, H_i)\hat{\alpha}^\top\right)$, and $B_i(\hat{\alpha}) = \frac{1}{\hat{\sigma_g^2}}(T_i, H_i)^\top (T_i, H_i)$. Note that (14) involves conditional expectations of cubic and quadratic terms of (T_i, H_i). It can be re-formulated in terms of the first and second conditional moments $\mu_i = \mathbb{E}[(T_i, H_i)]$, and $V_i = \mathbb{E}[(T_i, H_i)^\top (T_i, H_i)]$, which are readily available from the E step of EM algorithm,

$$\begin{aligned}\mathcal{I}(\hat{\alpha}) = \frac{1}{(\hat{\sigma_g^2})^2}\sum_{i=1}^{N}\Big\{ &\hat{\sigma_g^2} V_i - Z_i^2 V_i + Z_i\Big(\mu_i^\top \hat{\alpha} V_i + (\mu_i^\top \hat{\alpha} V_i)^\top + \hat{\alpha}\mu_i^\top (V_i - 2\mu_i^\top \mu_i)\Big) \\ &+ Z_i\Big(\mu_i^\top \hat{\alpha} V_i + (\mu_i^\top \hat{\alpha} V_i)^\top + \hat{\alpha}\mu_i^\top (V_i - 2\mu_i^\top \mu_i)\Big)^\top \\ &- 2V_i\hat{\alpha}^\top\hat{\alpha}V_i - \mu_i\hat{\alpha}^\top\hat{\alpha}\mu_i^\top(V_i - 2\mu_i^\top\mu_i) - \operatorname{tr}[\hat{\alpha}^\top\hat{\alpha}(V_i - \mu_i^\top\mu_i)]V_i \\ &- \sum_{j=1, j\neq i}^{N} (Z_i\mu_i^\top - V_i\hat{\alpha}^\top)(Z_j\mu_j^\top - V_j\hat{\alpha}^\top)^\top \Big\}.\end{aligned}$$

S 2 Simulation

In this section, we first show the the simulation results omitted in the main article. We then describe the additional simulations and show the corresponding results.

S 2.1 Simulation results of the main article

We evaluated the performance of GLM-PO2PLS for both normally and Bernoulli distributed outcome under different combinations of sample size, dimensionality, heterogeneity level, and noise level. In the main article, we only described the results of coefficient estimation and outcome prediction in high-dimensional settings. Here, we show the results of coefficient estimation, type I error and outcome prediction in low-dimensional settings, and the results of loading estimation and feature selection in both high and low dimensions.

Figure S1 depicts the results of coefficient estimation in low-dimensional settings. Overall, the performance was similar in low-dimensional and high-dimensional settings. In the scenarios with small sample size and high noise, the errors were larger than those in the high-dimensional settings.

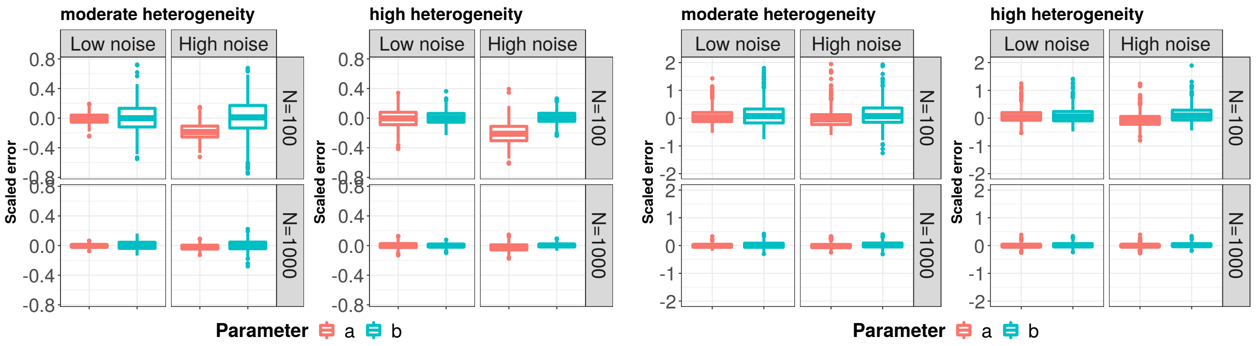

(a) Continuous z (b) Binary z

Figure S1: **Performance of coefficient estimation in low-dimensional settings.** The y-axis shows the scaled estimation error. Boxes show the results of 500 repetitions.

Figure S2 shows the type I error in low-dimensional settings. Overall, they were higher than those in the high-dimensional settings. In scenarios with small sample size and high noise level, the type I errors were above 10%. The type I errors decreased below 6% when the sample size was increased to 1000, and moved closer to 5% when the sample size further increased to 10000.

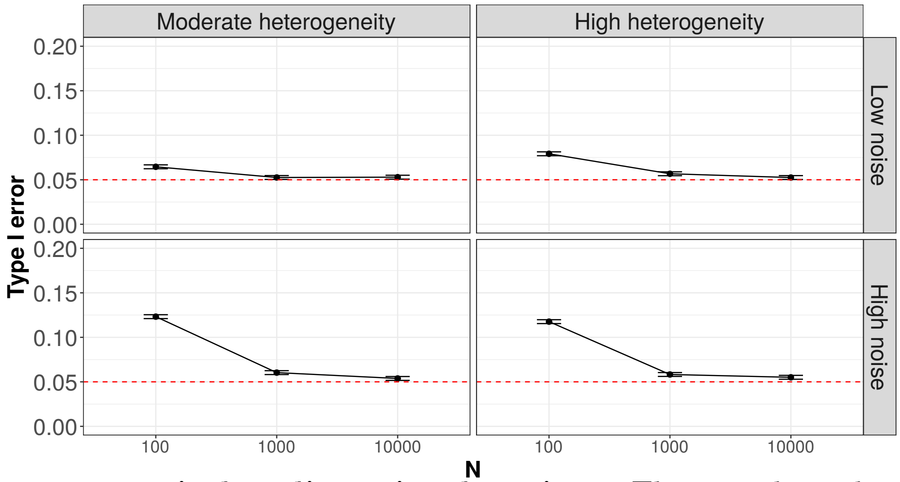

Figure S2: **Type I error in low-dimensional settings.** The error bars show the standard errors of the estimation. The dotted horizontal line is the significance level of 5%.

Figure S3 depicts the results of outcome prediction in low-dimensional settings. The performance was similar to that in the high-dimensional settings. The conclusions in high dimensional

settings in the main article also hold for low dimensional settings.

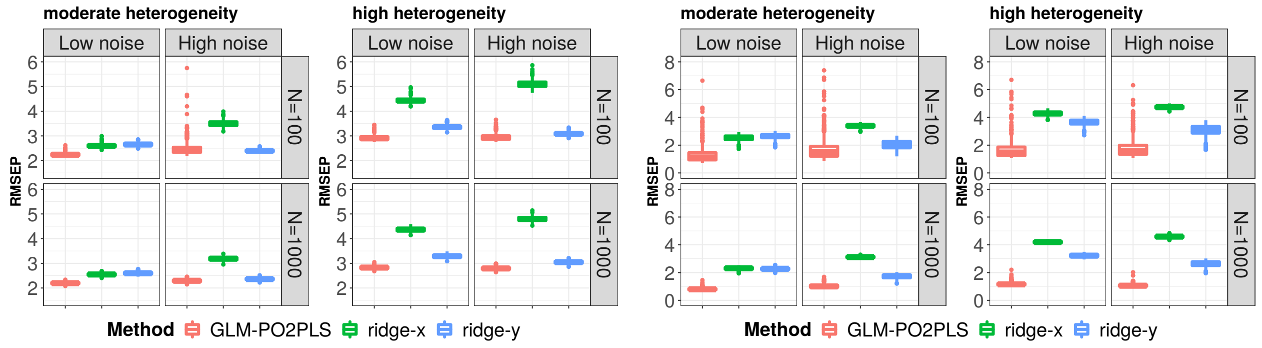

(a) Continuous z (b) Binary z

Figure S3: **Performance of outcome prediction in low-dimensional settings.** y-axis shows the root mean square error of prediction (RMSEP). Boxes show the results of 500 repetitions.

Figure S4 shows the inner products of the estimated loading vectors and the corresponding true loadings. Overall, the loading estimation was accurate except for the x-loadings in the scenarios of small sample size, high noise level. Be reminded that in these scenarios where the x-loadings were not well estimated, the coefficient a for the x-joint components in the linear predictor of z tended to be underestimated.

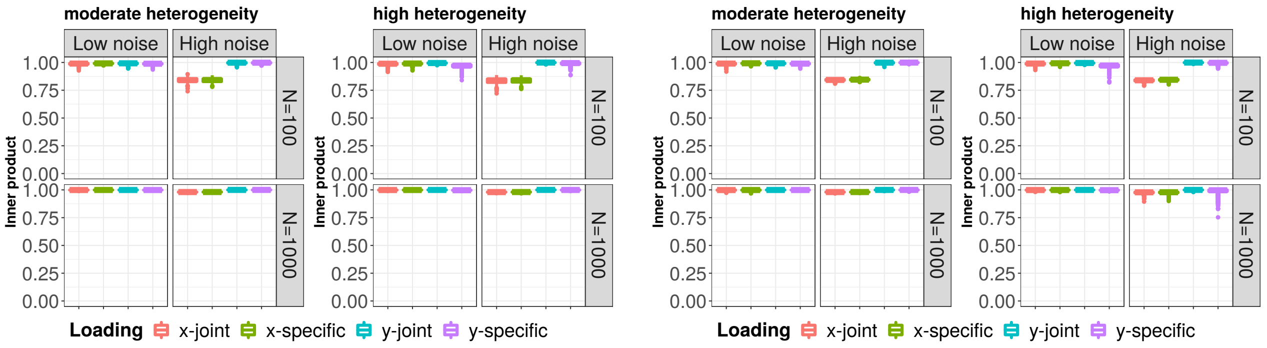

(a) Continuous z (high-dimensional) (b) Binary z (high-dimensional)

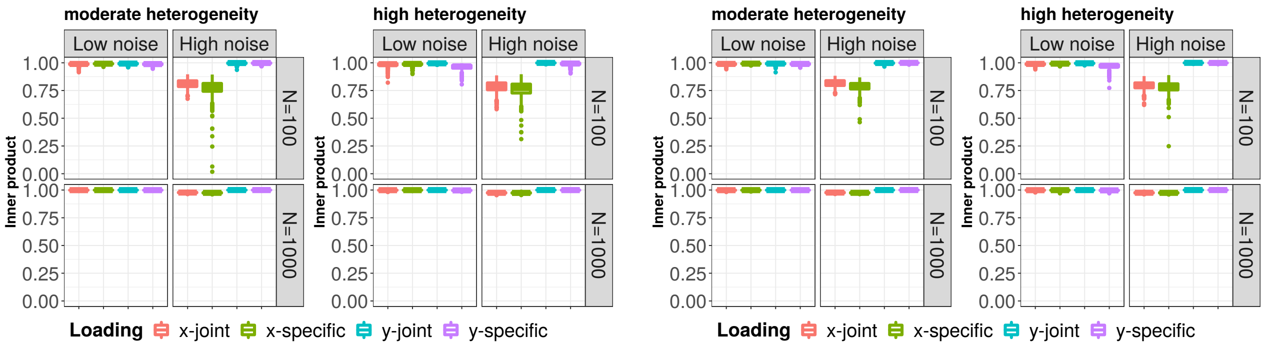

(c) Continuous z (low-dimensional) (d) Binary z (low-dimensional)

Figure S4: **Performance of loading estimation.** y-axis shows the inner product of the estimated loading vectors and the corresponding true loadings. Inner product of 1 suggests the loading vector is accurately estimated. Boxes show the results of 500 repetitions.

Figure S5 shows the results regarding performance of feature selection. From Figure S5a for continuous outcome in high-dimensional settings, in the scenarios with small sample size and moderate heterogeneity, the median TPR of GLM-PO2PLS was 0.90 under low noise level and

decreased to 0.62 when the dataset was more noisy. The TPR of ridge-x stayed around 0.25 regardless of the noise level. When the sample size was large, the median TPR of GLM-PO2PLS increased to 0.96 under low noise level and to 0.86 under high noise level. The median TPR of ridge-x increased to 0.50 in both noise levels. The amount of heterogeneity did not have much impact on the TPRs. Comparing Figure S5b for binary outcome to Figure S5a, GLM-PO2PLS performed similarly well, while the performance of ridge-x improved. In low dimensions (Figure S5c and Figure S5d), the TPRs of GLM-PO2PLS decreased slightly, compared to the TPRs in high-dimensional settings. On the contrary, the TPR of ridge-x increased substantially. Note that GLM-PO2PLS still outperformed ridge-x in low dimensions.

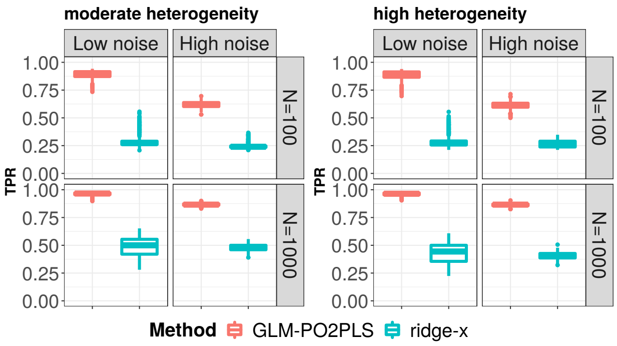

(a) Continuous z (high-dimensional)

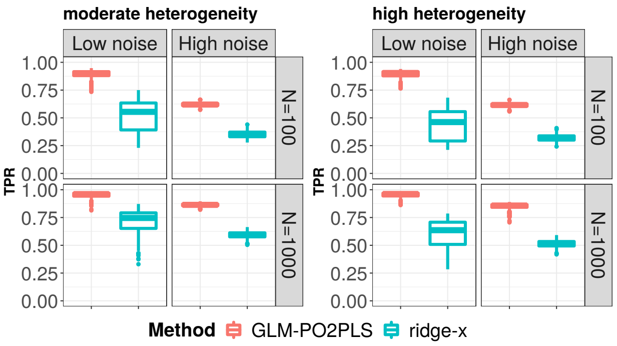

(b) Binary z (high-dimensional)

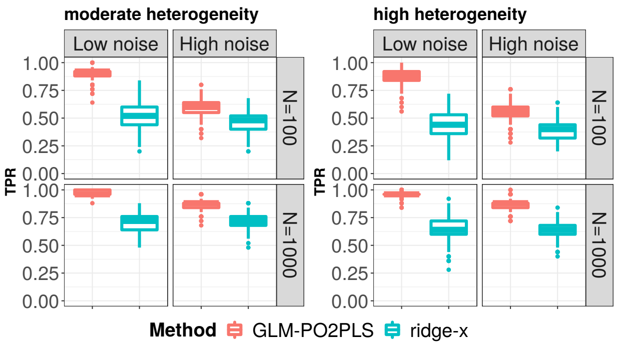

(c) Continuous z (low-dimensional)

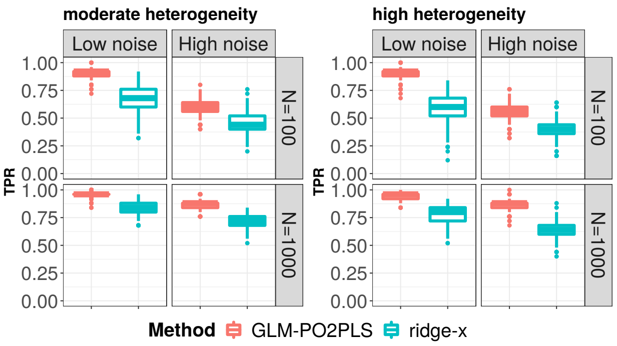

(d) Binary z (low-dimensional)

Figure S5: **Performance of feature selection.** y-axis shows the true positive rate calculated on the top 25% of features in x with the largest absolute loading values in GLM-PO2PLS, or with the largest absolute regression coefficients in ridge regression). Boxes show the results of 500 repetitions.

S 2.2 Additional simulations

We perform two additional simulations to evaluate the estimation accuracy of a and b with multiple components, and the power of the chi-square test using parameters estimated from the Down syndrome dataset. We consider here only high dimensional omics ($p = 2000$, $q = 25$) with a continuous outcome.

S 2.2.1 Coefficient estimation with multiple components

To investigate the coefficient estimation performance with multiple components, we set the number of joint, x-specific, and y-specific components to 2. The coefficient vectors $a = (a_1, a_2)$ and $b = (b_1, b_2)$ are set to $(2, 2)$ and $(1, 1)$, respectively. The other parameters are kept the same

as specified in Table 1 of the main manuscript. The errors $\hat{a_k} - a_k$ and $\hat{b_k} - b_k$ are standardized by a_k and b_k (where $k = 1, 2$) to exclude the influence of the parameter scale.

Figure S6 shows the scaled error of each coefficient. The errors of the first pair (a_1, b_1) were similar to the errors of the second pair (a_2, b_2). Compared to the results from a model with 1 pair of joint components, the estimation accuracy was similar.

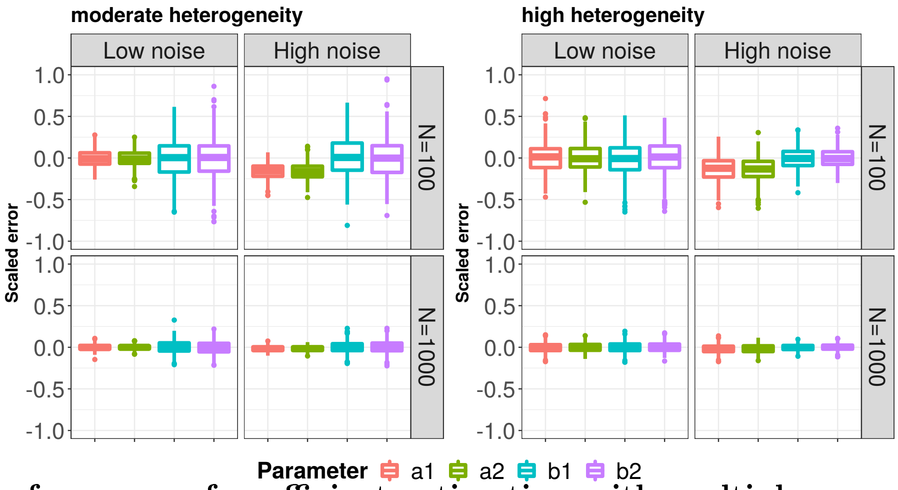

Figure S6: **Performance of coefficient estimation with multiple components.** The y-axis shows the scaled estimation error. In the moderate and high heterogeneity settings, h account for 40% and 80% of total variance in $u = tB + h$, respectively. Boxes show the results of 500 repetitions.

S 2.2.2 Power in settings estimated from dataset

We investigate the empirical power under settings estimated from the Down syndrome data. The noise levels are 80% in x, 20% in y, and 75% in z. The heterogeneity level (proportion of h in u) is 90%. The ratio of the explained variance by the x-specific part and x-joint part (i.e., $\sigma^2_{t_\perp}/\sigma^2_t$) is 2, and the ratio of y-specific part to y-joint ($\sigma^2_{u_\perp}/\sigma^2_u$) is 0.1. The coefficient a and b are set to 0.05 and 0.1, respectively. The matrix B is $0.04 \times I$. We investigate two sample sizes (100 and 1000), and four additional noise levels in z (60%, 90%, 95%, and 99%). 500 replications are simulated.

Figure S7 shows the empirical power under different noise levels in the outcome. Under a small sample size, the empirical power was 0.998 at the noise level of 75%, which was estimated from the Down syndrome data. The empirical power remained high at 0.862 with a higher noise level of 90%. With a large sample size of 1000, the empirical power was 1 with all the noise levels below 95% and dropped to 0.814 under an extreme noise level of 99%.

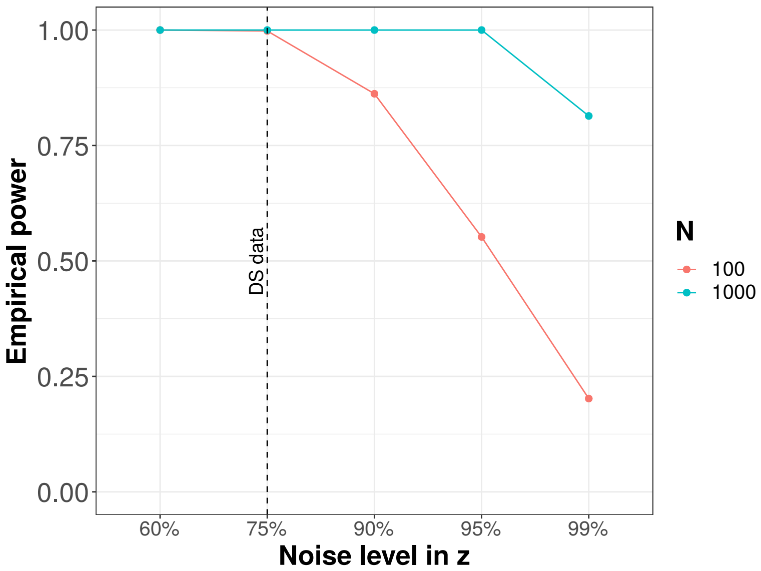

Figure S7: **Empirical power.** The noise proportions in the outcome z is shown on the x-axis, among which 75% is estimated from the DS dataset.

References

[1] Larry Armijo. Minimization of functions having lipschitz continuous first partial derivatives. *Pacific Journal of Mathematics*, 16(1):1–3, jan 1966.

[2] Said el Bouhaddani, Hae Won Uh, Caroline Hayward, Geurt Jongbloed, and Jeanine Houwing-Duistermaat. Probabilistic partial least squares model: Identifiability, estimation and application. *Journal of Multivariate Analysis*, 167:331–346, 2018.

[3] Said el Bouhaddani, Hae-Won Uh, Geurt Jongbloed, and Jeanine Houwing-Duistermaat. Statistical Integration of Heterogeneous Data with PO2PLS. mar 2021.

[4] David A. Harville. *Matrix Algebra From a Statistician's Perspective*. Springer New York, 1997.

[5] Gen Li and Sungkyu Jung. Incorporating covariates into integrated factor analysis of multi-view data. *Biometrics*, 73(4):1433–1442, 2017.

[6] A Woodbury Max. *Inverting modified matrices*. Princeton Univ., 1950.

[7] Xiao Li Meng and Donald B Rubin. Maximum likelihood estimation via the ecm algorithm: A general framework. *Biometrika*, 80(2):267–278, jun 1993.
